# Indocyanine Green Angiography as the Principal Design and Perfusion Assessment Tool for the Supraclavicular Artery Island Flap in Head and Neck Reconstruction

**DOI:** 10.7759/cureus.29007

**Published:** 2022-09-10

**Authors:** Jo-Lawrence M Bigcas, Carolyn A DeBiase, Tang Ho

**Affiliations:** 1 Otolaryngology - Head and Neck Surgery, University of Nevada Las Vegas School of Medicine, Las Vegas, USA; 2 Otorhinolaryngology - Head and Neck Surgery, Montefiore Medical Center, The Bronx, USA; 3 Otorhinolaryngology - Head and Neck Surgery, McGovern Medical School at UTHealth, Houston, USA

**Keywords:** perfusion, axial flaps, intraoperative imaging, head and neck reconstruction, supraclavicular artery island flap, indocyanine green angiography

## Abstract

A consecutive case series of supraclavicular artery island flaps was designed using indocyanine green angiography (IcG-A) in head and neck reconstruction to demonstrate its utilization in supraclavicular artery island flap (SCAIF) head and neck reconstruction. IcG-A was used consecutively between April 2014 and July 2015 to evaluate its use in flap design, inset, and intraoperative decision-making in five patients undergoing head and neck reconstruction. Six SCAIFs were harvested in five patients where IcG-A was used as the primary tool for flap design by visually mapping the supraclavicular artery under fluorescence. Each flap was harvested around the mapped course of the artery. Confirmatory Doppler was present in each flap raised with this technique. In all five patients, IcG-A was used to assess flap perfusion after inset. This case series demonstrates IcG-A as another tool for SCAIF design in head and neck reconstruction. The technology provides direct visualization of the pedicle before harvest. It can also be used as an intraoperative tool to visualize the blood supply once the flap is rotated to assess flap perfusion and detect areas that may be compromised, thereby improving flap survival.

## Introduction

Head and neck reconstruction employs the entire spectrum of the reconstructive ladder. In patients where a free microsurgical flap may not be an option, a pedicled flap is an alternative option. Where in the past we may have considered a pectoralis flap or a free microvascular flap, the supraclavicular artery island flap (SCAIF) has gained popularity over the years for its versatility, good esthetic results of both the donor and recipient sites, short operating time, and relative ease of technique compared to free flaps.

The vascular pedicle of the SCAIF arises from the transverse cervical artery with two accompanying veins. It is described as a fasciocutaneous pedicled flap accompanied by the supraclavicular artery with a subcutaneous pedicle measuring up to 20 cm. When rotated around its pivot point, it can be used to cover defects in the chest, neck, chin, and cheek. In our experience, it can reach as high as the parietal scalp.

This series focuses on the use of indocyanine green (IcG) fluorescent angiography in SCAIFs for head and neck reconstruction. IcG is a fluorescent marker that binds plasma proteins and is subsequently excreted through the bile with a half-life of 3-4 min. It has a well-known safety profile and toxicity is rare [1-2]. IcG was originally Food and Drug Administration (FDA)-approved to assess coronary artery bypass graft patency [3]. Its use has extended to many areas including sentinel lymph node biopsy, assessment of free flaps especially in breast reconstruction, evaluation of lymphedema, and traumatic injuries [1, 4-10]. Studies have assessed the use of IcG-A in pedicled flaps and have been met with promising results [11-14]. In a prospective series of pedicled skin flaps, intraoperative IcG filling defects correlated with delayed wound healing, whereas flaps without IcG-A filling defects all healed primarily [12]. Few studies to date have been dedicated to the integrated use of IcG-A with the SCAIF in head and neck reconstruction. This study explores the benefits of using IcG-A intraoperatively during a SCAIF procedure.

## Case presentation

IRB approval

The study was approved by The University of Texas Institutional Board Review, IRB HSC-MS-15-0728. Prior to surgery, patients were informed that IcG-A would be used to map the course of the supraclavicular artery. Charts were reviewed retrospectively, and data were compiled for publication purposes.

Surgical method

**Figure 1:**
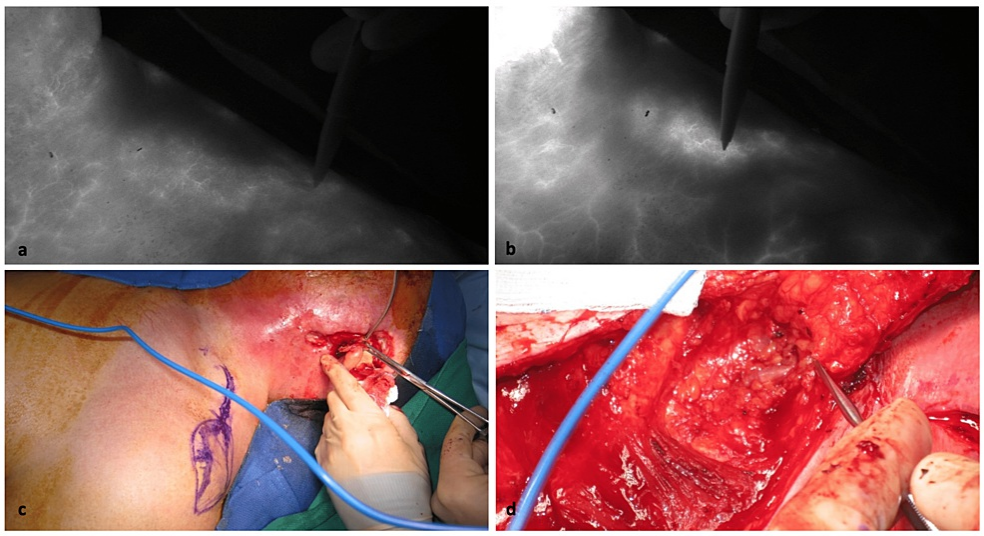
Intraoperative photos from Case 1. (a) Pen tracing of supraclavicular artery under fluorescence after initial IcG injection. (b) Pen tracing more distal supraclavicular artery after full perfusion of IcG. (c) Intraoperative planning of flap shown on skin and resection defect visible. (d) Raised flap with forceps pointing towards the vascular pedicle. IcG, indocyanine green

Indocyanine green was prepared by diluting IcG to a concentration of 2.5 mg/mL. Prior to harvest planning, IcG was injected intravenously and imaged using the SPY Elite Fluorescent Imaging System (Stryker, Kalamazoo, MI). Fluorescent light is used to identify the course of the supraclavicular artery, which is marked on the skin (Figure 1a-b). The SCAIF was designed around the marked supraclavicular artery (Figure 1c). The flap was harvested in a soft fascial manner in a distal to proximal manner around the course of the supraclavicular artery (Figure 1d). After harvest, the pedicle is confirmed with Doppler. The pedicle is then rotated and inset. Confirmatory injection of IcG is then used to ascertain preservation of the pedicle after rotation and inset under fluorescent light. Areas of poor perfusion on confirmatory IcG-A would be addressed by adjusting the flap (e.g., relieving stress caused by flap rotation, removal of areas of poor perfusion, or abandoning the flap in favor of an alternative reconstruction plan). 

Results

Using the method described earlier, IcG-A was used as the principal instrument in designing six consecutive SCAIFs in five patients between 2014 and 2015. Four out of the five patients were post-ablative cancer patients; one patient was a complicated post-traumatic wound. Post-harvest confirmatory Doppler was found in all six flaps. Five out of these six flaps were rotated and inset. IcG-A was used in five out of five rotated flaps after inset. Perfusion was confirmed in all five flaps. A summary of the case series is described in Table 1. Specific details are described on a case-by-case basis below.

**Table 1:** Summary of the series. All flaps were designed using IcG-A. All flaps had Doppler signal present after harvest. In each case, IcG-A was used to assess perfusion after inset. IcG-A, indocyanine green angiography; SCAIF, supraclavicular artery island flap; GSW, gun shot wound; BMI, body mass index

Case	Age/Sex	Cause of defect	Size of defect (cm)	Defect location	Flap size (cm)	Complications
1	81/M	Recurrent malignancy, prior radiation	5 x 3	Left total auricle, mastoid, neck, parietal bone	20 x 5	None
2	44/F	Trauma, GSW	4 x 5	Right mandibular parasymphysis and body, orocutaneous fistula	15 x 5	None
3	79/M	Malignancy	6 x 7	Left cheek skin	15 x 4	None
4	55/F	Malignancy	Intraoral: 5 x 6 Skin: 4 x 3	Right buccal mucosa and cheek skin	15 x 5	No complications with SCAIF. Dehiscence of cervicofacial rearrangement of the skin wound, managed with wound care.
5	66/M	Malignancy	4 x 3	Oral cavity composite resection, floor of mouth, mandible, mucosal lip	15 x 4	Attempted right-sided osteofasciocutaneous flap. Flap aborted due to unfavorable geometry. Wound breakdown of closure and clavicular plate exposure at donor site.
					15 x 3	Wound breakdown at donor site closure. Distal flap failure at recipient site. Wound healing issues due to malnutrition (BMI = 13), hypothyroidism.

Case 1

An 81-year-old male with a history of recurrent melanoma and squamous cell carcinoma of the left auricle and parotid underwent a prior wide local excision of a left preauricular melanoma followed by adjuvant radiation at an outside facility. He had cortical mastoid exposure from his radiation. He subsequently developed another auricular lesion, consistent with squamous cell carcinoma, and underwent salvage surgery, which included wide local excision and parotidectomy. The soft tissue defect measured 5 cm x 3 cm with a superior extent up to the parietal scalp. There have been no reports of the use of this flap as high as the parietal scalp. Due to the scarcity of literature detailing use of a SCAIF for scalp coverage, the plan was to use IcG-A to confirm adequate perfusion of the SCAIF after rotation and inset. 

**Figure 2:**
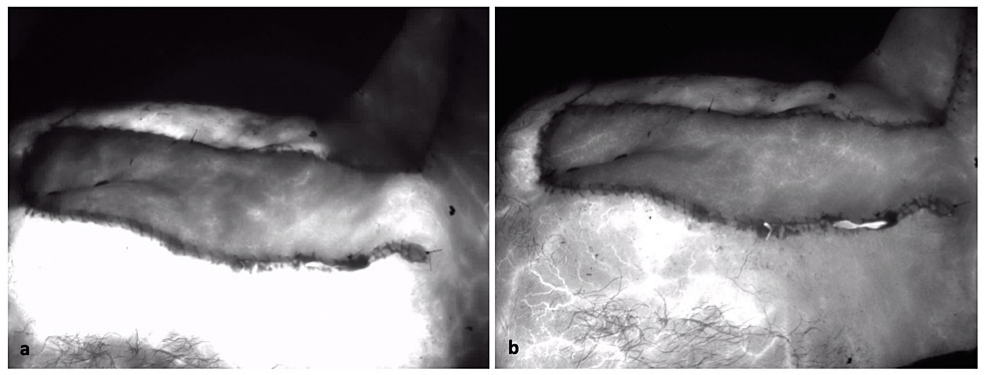
Intraoperative fluorescence imaging after inset of the flap. (a) Flap inset after infusion of indocyanine green. (b) Flap inset after IcG fully distributed. Note perfusion persists to distal end of flap.

Intraoperative IcG-A was used to map the course of the supraclavicular artery. A 20-cm SCAIF with a 10 cm x 5 cm skin island was harvested, rotated, and inset for full coverage of his wound. Confirmatory IcG-A was performed after inset to the parietal scalp, demonstrating adequate perfusion of the flap without the need for further revision (Figure 2). He had an uncomplicated postoperative course without flap dehiscence or compromise.

Case 2

A 44-year-old male, status post gunshot wound to the mandible, who underwent free scapular tissue transfer for reconstruction, developed a complicated draining right-sided orocutaneous fistula and a soft tissue defect of the submental area and the hardware stabilizing his reconstruction. The wound was refractory to local tissue debridement, soft tissue rearrangement, and wound care. He had an open wound of this mandible and submentum measuring approximately 4 cm x 5 cm. He underwent removal of hardware, excision of the fistula, and tissue debridement. The wound was reconstructed with an ipsilateral SCAIF. IcG-A was used during the procedure to identify the course of supraclavicular artery, around which a 15 cm x 5 cm flap was designed. Once again, confirmatory angiography was performed after inset. No distal ischemia was identified; thus, no revision of the flap was required after rotation. The wound healed well, and he had no further issues.

Case 3

A 79-year-old male with squamous cell carcinoma of the left cheek underwent wide local excision, left parotidectomy, and left neck dissection. The resulting skin defect was 6 cm x 7 cm along with a superficial parotidectomy defect on the deep aspect. IcG-A was used to design an ipsilateral 15 cm x 4 cm SCAIF to extend to the left cheek. Confirmatory angiography was performed after inset. The procedure was tolerated well. Post-operatively, he underwent chemoradiation and has had no complications associated with his flap.

Case 4

A 55-year-old female with a right buccal mucosal squamous cell carcinoma with extension toward the skin and anterior parotid. She underwent wide local excision, right parotidectomy, and bilateral neck dissection. The primary cancer extirpation resulted in a through-and-through 5 cm x 6 cm intraoral defect and a 4 cm x 3 cm skin defect. Using IcG-A, a SCAIF was designed along the course of the mapped supraclavicular artery. SCAIF was designed to measure 15 cm x 5 cm to close the intraoral defect. Local tissue rearrangement with a note flap was used to cover the skin defect. Confirmatory IcG-A was used after inset. No areas of ischemia were identified. The flap was recontoured solely to accommodate the shape of the intraoral wound. One month postoperatively, the patient developed some dehiscence of the skin portion of the wound (unrelated to the SCAIF) which was managed with local wound care during her adjuvant radiation course. The wound subsequently healed completely by four months after surgery (two months after completion of radiation therapy).

Case 5

A 66-year-old with a floor of mouth squamous cell carcinoma underwent floor of mouth excision, composite mandibular resection, and bilateral neck dissection, which resulted in a floor of mouth and segmental mandibular defect. The resulting wound was approximately 4 cm x 3 cm. The reconstructive plan was to create an osteofasciocutaneous SCAIF. Intraoperative IcG-A was performed to design a right-sided SCAIF inclusive of a right clavicular bone graft to be inset into the mandibular defect. Flap dimensions were 15 cm x 4 cm. Unfortunately, the geometry of the osseous SCAIF was unfavorable. The bone graft was separated from the flap and used as a free, non-vascularized graft. The right-sided SCAIF was laid back down. A larger, more geometrically favorable, contralateral SCAIF was raised to provide a vascular wrap on the bone graft. Again, intraoperative IcG-A was used to design a 15 cm x 3 cm SCAIF mapped according to the course of the supraclavicular artery. The clavicular bone graft was inset and fixated with two four-hole 1.5-mm tension bands. The SCAIF was used to cover the graft, hardware, and soft tissue defect. Confirmatory IcG-A was performed after inset, revealing a well-perfused flap. Overall, the procedure was well tolerated. Unfortunately, the patient experienced wound healing issues including clavicular plate exposure, flap dehiscence, orocutaneous fistula, and distal flap failure. While IcG-A demonstrated vascular coverage within the flap, other patient factors likely affected this patient’s post-operative course, including malnutrition (body mass index, BMI = 13) and hypothyroidism.

## Discussion

From its conception in 1949 to its multi-functional applications today, the supraclavicular flap has made considerable progress in surgical history. Kazanjian & Converse were the first physicians to use a shoulder flap, the “in charretera” flap [15]. Their innovation was further refined by two groups in 1979: Mathes & Vasconez, and Lamberty & Cormack. The introduction of the flap was met with some backlash due to unpredictable flap survival [16]. Lamberty addressed these concerns and clarified the vascularity and need to not inadvertently divide the supraclavicular artery while raising the flap [17].

The flap remained controversial despite the work of its predecessors until interest in the technique was reignited by Pallua in 1997 with his introduction of the use of the supraclavicular flap for postburn mentosternal contractures [18]. He went on to describe the use of the flap for more extensive head and neck defects, the tunneling technique of the flap, and full facial reconstruction [19-21]. The use of the SCAIF has been extended to use in head and neck oncologic defects including pharyngeal, tracheal-stomal, mandible, parotid, and irradiated neck defects [22-27]. In a study by Sheriff et al., they reviewed 25 SCAIFs and found that the risk for distal necrosis increases in flaps harvested at 23 cm or longer [28].

The IcG-A is increasing in use for intraoperative decision-making. In a publication by Lee et al. in 2014, intraoperative IcG-A used in two highly comorbid patients altered the reconstruction plan into a staged procedure using the vascular delay technique [29]. In a meta-analysis of IcG-A in breast reconstruction, the use of intraoperative angiography resulted in a statistically lower incidence of flap necrosis and reoperations [30]. The limitations of intraoperative angiography are that it requires a means for fluorescence imaging and IV contrast is administered. Indocyanine green is commonly administered in ophthalmology. In a study by Obana et al. in 1996, they reviewed 3774 IcG-A studies in 2820 patients at a dosage of 25-75 mg. Some 13 of the 3774 IcG-A studies described adverse reactions to the contrast. One patient experienced severe pain in the vein, which required treatment, and two patients required treatment for hypotension. The other 10 reactions were urticaria, itching, nausea, and urge to defecate [31].

The recent rise in popularity of the SCAIF can be attributed to its versatility, good esthetic results of both donor and recipient sites, shorter operating time, and relative ease of technique compared to free flaps. As Shenoy noted, the supraclavicular flap has the reliability and ease of harvest of a regional flap combined with the pliability and good color match normally seen in free flaps [25]. The location and versatility of the flap lend themselves to patients with previously irradiated or operated necks in head and neck cancer patients [25]. 

While intraoperative angiography and its applications have been extensively published, few studies are dedicated to its specific application in SCAIF head and neck reconstruction. In another study by Sheriff et al., they examined six different techniques for mapping the supraclavicular artery in 65 patients. Of these patients, eight patients were in the IcG-A arm, and one of those eight patients was a head and neck reconstruction patient. Their study found that IcG-A identified the pedicle and its partial course 60% of the time [32]. Another recent study dedicated to IcG-A in supraclavicular artery flaps was by Suzuki et al. Their study included six patients, five of which underwent head and neck reconstruction. Their study was focused on the use of IcG-A to assess perfusion for the thoracic branch variant of the supraclavicular artery island flap (TBSA), which places more of the skin paddle on the chest. Of the six flaps raised, one was a conventional SCAIF, four were TBSA, and one was a hybrid SCAIF/TBSA. In this study, IcG-A was employed after the flap was raised to assess vascularity and to remove areas void of fluorescence. Flaps ranged from 13 cm x 5.5 cm to 17 cm x 6.5 cm. They experienced no flap necrosis [33].

Our study demonstrates that IcG-A can be used as the principal tool in the design and perfusion assessment of the SCAIF for head and neck reconstruction. Our pilot patient (Case 1) had a prior history of surgery, radiation, and a wound extending onto the parietal scalp. Because no literature had ever detailed use of the SCAIF as high as the parietal scalp, IcG-A was selected to provide intraoperative perfusion assessment after flap rotation and inset. Angiography provided an additional level of confidence in planning flap boundaries and determining flap viability that Doppler alone could not. That patient experienced no complications postoperatively. The application of the technology was so inspiring, we continued to apply IcG-A on a consecutive series of SCAIFs, though the defects in remaining cases did not stress the flap as much as the pilot case.

All flaps designed and raised using IcG-A had confirmatory Dopplers. After rotation and inset, confirmatory IcG-A demonstrated ample flap perfusion, and the only revision made to a flap was for contouring to a wound defect, not distal ischemia. In our two later cases, both patients experienced complications. In one case, the SCAIF used to close the intraoral wound did not have complications, but rather the extraoral cervicofacial rearrangement. In the other patient, despite IcG demonstrating good flap perfusion, the patient experienced poor wound healing, likely secondary to malnutrition and hypothyroidism. He had bilateral donor site wound breakdown, as well as recipient site distal flap failure.

Considering the supraclavicular flap’s history of indeterminate reliability, IcG-A is a safe technology that may play a role in improving surgeon confidence in their reconstruction. This study shows that a SCAIF can be designed and raised with IcG-A. Intraoperative angiography can provide an added layer of confidence after rotation and inset. Flap failure can be avoided with judicious design and sound intraoperative judgment. Despite sound decision-making, patient factors can still affect successful, complication-free reconstruction.

## Conclusions

The SCAIF’s lack of popularity is largely from its variable results in different surgeons' hands, as well as some of its perceived limitations on how far the flap can reach. Our case series includes a SCAIF that was able to provide coverage on the parietal scalp. There is sparse literature on SCAIFs having this type of reach. IcG-A was used after flap rotation and inset to demonstrate that this flap can feasibly cover parts of the scalp.

Our case series demonstrates the use of IcG-A as a potentially powerful primary tool in the planning and implementation of the supraclavicular artery island flap for head and neck reconstruction. The standard technique of designing SCAIFs includes mapping the course of the supraclavicular artery and letting primary closure dictate the optimal flap harvest while minimizing morbidity. IcG-A can provide complementary information about patterns of perfusion to avoid flap failure. 
